# Supplementary material for: Stable Luminescent Poly(Allylaminehydrochloride)-Templated Copper Nanoclusters for Selectively Turn-Off Sensing of Deferasirox in β-Thalassemia Plasma
Source: Pharmaceuticals (Basel). 2021 Dec 16;14(12):1314. doi: 10.3390/ph14121314 (PMC8706525; doi:10.3390/ph14121314)
Supplement: Supplementary file 1 [file pharmaceuticals-14-01314-s001.zip › pharmaceuticals-1492294-supplementary.pdf]

Stable Luminescent Poly(Allylaminehydrochloride)-Templated Copper Nanoclusters for Selectively Turn-Off Sensing of Deferasirox in β-Thalassemia Plasma

Hung-Ju Lin [1], Chun-Chi Wang [1,2,3,*], Hwang-Shang Kou [1], Cheng-Wei Cheng [1] and Shou-Mei Wu [4,5,*]

[1] School of Pharmacy, College of Pharmacy, Kaohsiung Medical University, Kaohsiung 807, Taiwan; Weiting0825@gmail.com (H.-J.L.); kouhs@kmu.edu.tw (H.-S.K.); pharmacysniksam@yahoo.com.tw (C.-W.C.)
[2] Department of Medical Research, Kaohsiung Medical University Hospital, Kaohsiung 807, Taiwan
[3] Drug Development and Value Creation Research Center, Kaohsiung Medical University Hospital, Kaohsiung 807, Taiwan
[4] Department of Fragrance and Cosmetic Science, College of Pharmacy, Kaohsiung Medical University, Kaohsiung 807, Taiwan
[5] Taiwan Food and Drug Administration, Ministry of Health and Welfare, Taipei 11561, Taiwan
* Correspondence: chunchi0716@kmu.edu.tw (C.-C.W.); shmewu@kmu.edu.tw (S.-M.W.); Tel.: +886-7-3121101 (C.-C.W.); +886-7-3121101 (S.-M.W.)

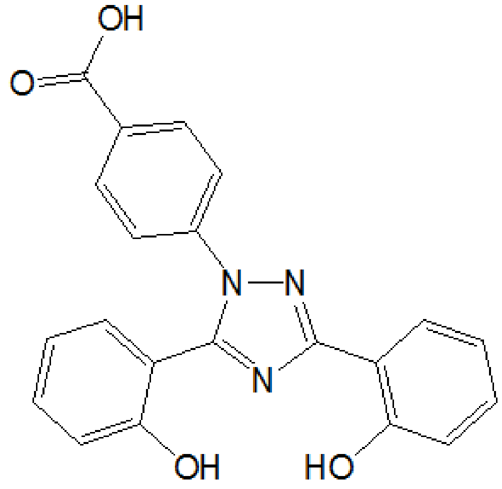

Figure S1. Chemical structure of deferasirox.

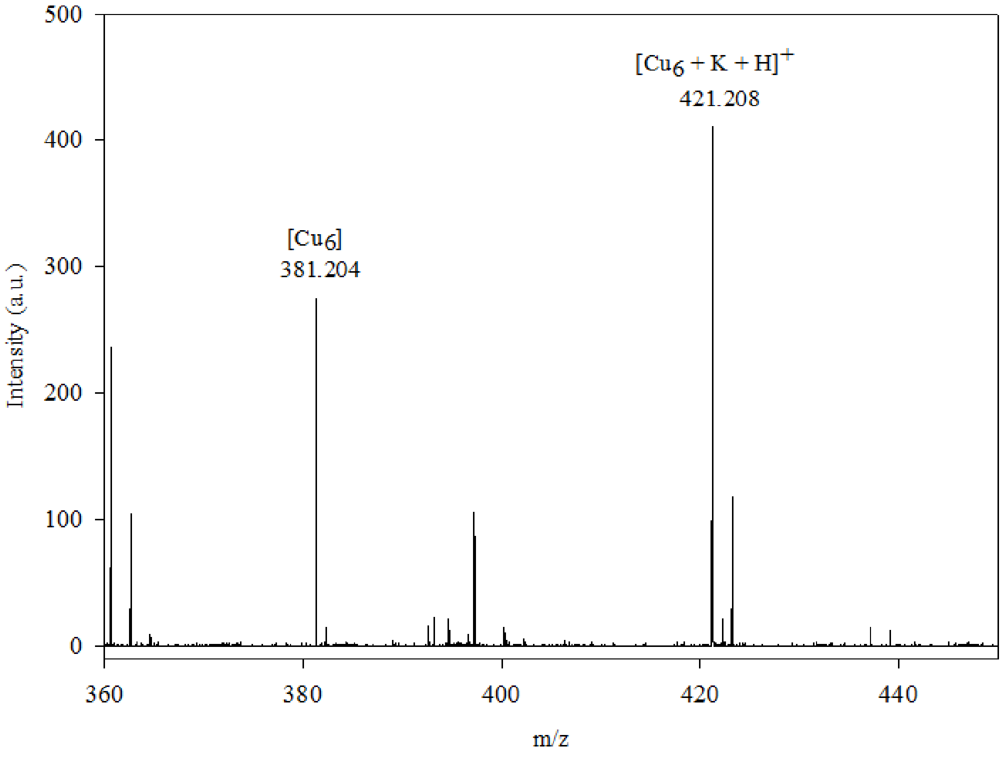

Figure S2. The MALDI-MS spectrum of PAH-Cu NCs.

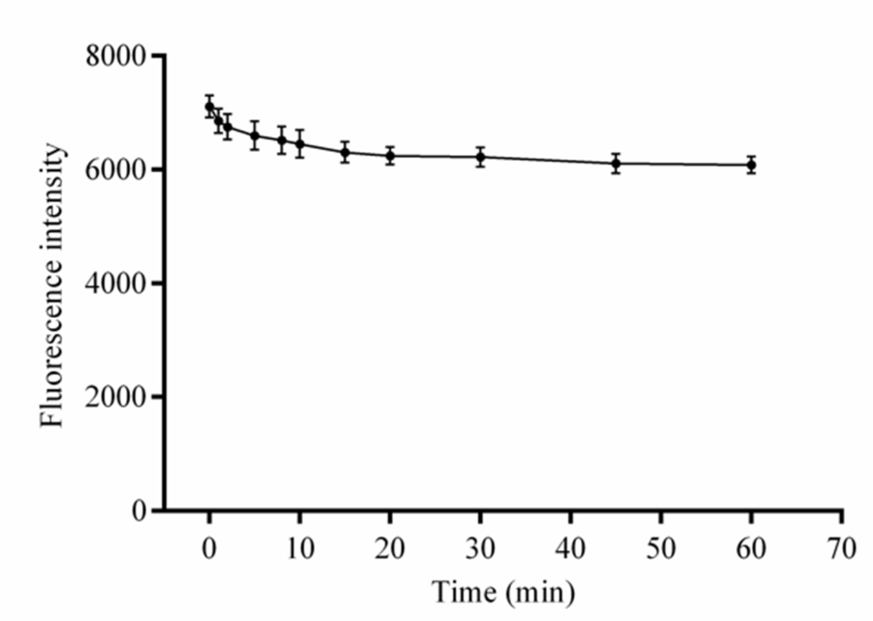

Figure S3. Fluorescent stability of the PAH-Cu NCs was evaluated for 60 min with continuously exposing at excitation source (360 nm).
